# RETRACTION: The Connotation Between Perioperative Glycemic Control Approach and Sternal Wound Infection in Individuals With Diabetes Mellitus Experiencing Cardiac Surgery: A Meta‐Analysis

**DOI:** 10.1111/iwj.70209

**Published:** 2025-02-02

## Abstract

**RETRACTION:** TanG. , LiY. , and ZhouG. , “The Connotation Between Perioperative Glycemic Control Approach and Sternal Wound Infection in Individuals With Diabetes Mellitus Experiencing Cardiac Surgery: A Meta‐Analysis,” International Wound Journal 20, no. 8 (2023): 3324–3330, 10.1111/iwj.14213.37190865 PMC10502249

The above article, published online on 15 May 2023, in Wiley Online Library (http://onlinelibrary.wiley.com/), has been retracted by agreement between the journal Editor in Chief, Professor Keith Harding; and John Wiley & Sons Ltd. Following an investigation by the publisher, all parties have concluded that this article was accepted solely on the basis of a compromised peer review process. The editors have therefore decided to retract the article. The authors did not respond to our notice regarding the retraction.

**RETRACTION:** G. Tan , Y. Li , and G. Zhou , “The Connotation Between Perioperative Glycemic Control Approach and Sternal Wound Infection in Individuals With Diabetes Mellitus Experiencing Cardiac Surgery: A Meta‐Analysis,” International Wound Journal 20, no. 8 (2023): 3324–3330, 10.1111/iwj.14213.37190865 PMC10502249

The above article, published online on 15 May 2023, in Wiley Online Library (http://onlinelibrary.wiley.com/), has been retracted by agreement between the journal Editor in Chief, Professor Keith Harding; and John Wiley & Sons Ltd. Following an investigation by the publisher, all parties have concluded that this article was accepted solely on the basis of a compromised peer review process. The editors have therefore decided to retract the article. The authors did not respond to our notice regarding the retraction.

